# MicroRNA miR-30a inhibits cisplatin resistance in ovarian cancer cells through autophagy

**DOI:** 10.1080/21655979.2021.2001989

**Published:** 2021-12-10

**Authors:** Yi Cai, Baiping An, Dejiao Yao, Hong Zhou, Jie Zhu

**Affiliations:** Department of Oncology, Hospital of Chengdu University of Traditional Chinese Medicine, Chengdu, China

**Keywords:** miR-30a, Sensitivity, Ovarian cancer, Cisplatin, Autophagy

## Abstract

We study whether microRNA miR-30a inhibits the autophagy through transforming growth factor (TGF)-β/Smad4 to generate cisplatin (DDP) resistance in ovarian cancer cells. The expression of miR-30a, Smad4, and TGF-β was detected in the serum of ovarian cancer patients and DDP-resistant cell lines (A2780) by quantitative real-time polymerase chain reaction (qRT-PCR). Computational search and western blotting were used to demonstrate the downstream target of miR-30a in ovarian cancer cells. Cell viability was measured with CCK8 assay. Apoptosis and autophagy of ovarian cancer cells were analyzed by flow cytometry and transmission electron microscopy, and the expressions of Beclin1 and LC3II protein were detected by western blotting. Expression of miR-30a was significantly decreased, while expressions of TGF-β and Smad4 mRNA were increased in serum of ovarian cancer patients after DDP chemotherapy as well as in DDP-resistant cells. Activation of autophagy contributed to DDP-resistance cells. Moreover, Bioinformatics software predicted Smad4 to be a target of miR-30a. Overexpression of miR-30a decreased the expression of Smad4 and TGF-β. Additionally, miR-30a-overexpressing inhibited DDP-induce autophagy and promoted DDP-resistant cell apoptosis. In conclusion, miR-30a mediates DDP resistance in ovarian cancer by inhibiting autophagy via the TGF-β/Smad4 pathway.

## Introduction

Ovarian cancer (OC) is the seventh most common cancer among women in the world [1]. In 2020 worldwide, around 312,800 women have been diagnosed with OC and around 206,800 have died of this disease [2]. Based on the TNM staging classification of the 6th edition of the American Cancer Joint Committee (AJCC), the 5-year survival rates of OC patients with stages I–II and III–IV are 60−75%, and 19–41%, respectively [3]. However, 70% of OC patients present with metastasis at diagnosis, 80−90% of whom have already missed the surgical opportunity [4]. Therefore, chemotherapy is indispensable in the treatment of OC [5]. At present, Cisplatin (DDP)-based chemotherapy is an essential approach to treat the ovarian cancer. However, the 5-year survival rate of OC patients is less than 30% due to the resistance of OC cells to DDP [6]. Therefore, the overall efficacy of DDP is not ideal in the clinical practice, and it is necessary to understand the mechanisms of drug resistance. Autophagy is the main pathway by which the body removes damaged, senescent, degenerated, and nonfunctional proteins and organelles [7]. Autophagy is a self-protective mechanism that can lead to cell death during emergency responses [8]. Studies have shown that autophagy is closely related to the development of tumors [9] and its self-protective ability increases the resistance of cancer cells to chemotherapeutic agents [10]. In recent years, many studies have shown that autophagy is associated with chemoresistance in ovarian cancer. Zhou et al. [11] showed that downregulation of OGT enhanced cisplatin-induced autophagy, which led to cisplatin-resistant ovarian cancer. Hu et al. [12] reported that miR-29 c-3p overexpression attenuated DDP resistance by inhibiting autophagy. These studies confirm that inhibition of autophagy reduces cisplatin resistance in OC cells.

MicroRNAs (miRNAs) are a group of small, non-coding RNAs in eukaryotes with 20−25 nucleotides in length, involved in the post-transcriptional regulation of genes [13]. The highly conserved miRNAs greatly regulate the body. Recent studies have confirmed the close relationship between miR-30a and the pathogenesis, progression and drug resistance mechanisms of tumors [14–16]. Another study has reported that miR-30a regulates the chemotherapeutic resistance of tumor cells by regulating the level of autophagy in tumor cells [17]. MiR-30a can enhance the sensitivity of gastric cancer cells to cisplatin by inhibiting the epithelial-mesenchymal transition (EMT) [18]. MiR-30a-5p can affect the resistance of melanoma cells to cisplatin by targeting the IGF1R gene [19]. In addition, Sestito et al. showed that the overexpression of miR-30a can provide the sensitivity of epithelial OC cells to cisplatin by inhibiting EMT [20]. It was also found that increasing miR-30a expression inhibited DDP-induced autophagy in non-small cell lung cancer (NSCLC) cells, thereby disrupting the resistance of NSCLC cells to DDP [21]. However, no study has reported whether miR-30a affects the resistance of OC to DDP by regulating cellular autophagy. Based on the above studies, we speculate that miR-30a may be involved in regulating chemoresistance in OC cells through the inhibition of cellular autophagy. Therefore, the present study aimed to investigate the role of miR-30a expression in autophagy and DDP resistance in OC.

## Materials and methods

### Subjects

A total of 47 patients with ovarian cancer who underwent radical surgery and received the same DDP-based combination chemotherapeutic regimen at our hospital were retrospectively collected. The average age was 61 years (ranging from 48 to 75). All patients were pathologically confirmed with ovarian cancer. Blood samples were collected before chemotherapy and 24 h after chemotherapy, after obtaining informed consent. This study was approved by the Ethics Review Committee of Affiliated Hospital of Chengdu University of Traditional Chinese Medicine (Approval number: 2020KBL-018).

### Materials, reagents and apparatus

PCR primer, has-miR-30a mimics, and mimics NC were purchased from Genepharma, Shanghai. TRIzol, Lipofectamine^TM^ 2000, donkey anti-mouse Alexa Fluor680 secondary antibody, and donkey anti-rabbit Alexa Fluor 680 secondary antibody were purchased from Invitrogen, USA. Reverse transcription of PCR was performed by the High Capacity cDNA Reverse Transcription kit (Applied Biosystems, USA), and the mir Vana miRNA isolation kit was purchased for isolation and purification of miRNA (Applied Biosystems, USA). The primary antibodies against LC3, Beclin1, TGF-β, Smad4, and β-actin were purchased from Santa Cruz, USA. Cell Counting Kit (CCK-8) was bought from Boster, Wuhan. Monodansyl cadaverine (MDC) and autophagy inhibitor, 3-MA, were both purchased from Sigma, USA. Apoptosis kit was purchased from Becton Dickinson, USA. Autophagy activator rapamycin was purchased from CST, USA. Also, human ovarian cancer cell line A2780 was obtained from the Cell Bank of Chinese Academy of Sciences.

### Cell culture

Ovarian cancer cells A2780 and A2780 DDP-resistant cells (A2780-DDP) were purchased from the American Type Culture Collection (ATCC, USA) and cultured in the RPMI1640 medium (Gibco, USA) supplemented with 10% fetal bovine serum (FBS, Gibco, USA) and 100 U/mL penicillin and 100 U/mL streptomycin at 37°C in a 5% CO_2_ incubator. A2780-DDP cells were incubated in complete medium containing 2 μmol/L DDP to maintain the resistance [22]. Follow-up experiments were performed when the cells were passed to the third generation.

### Quantitative real-time PCR (qRT-PCR)

Total RNA was extracted from ovarian cancer cell line and patient blood using TRIzol kit, followed by reverse transcription into cDNA according to the manufacturer’s instructions. Primers for Smad4, TGF-β, β-actin, miR-30a, and U6 genes were designed and synthesized for qRT-PCR amplification (β-actin was utilized as an internal control for the mRNA expression, while U6 gene was utilized as an internal control for miR-30a expression). The primer sequences were designed and synthesized by Sangon (Shanghai) Biotech Co., Ltd. (China), and the primer sequences are shown in Table 1. The specific procedure was performed according to the manufacturer’s instructions. The relative expression of the target gene was calculated using the 2^−ΔΔCT^ method.

**Table 1.** Primer sequences

Gene	Primer sequences
Smad4	Forward: 5ʹ-AGGATCAGTAGGTGGAATAG-3’
	Reverse: 5ʹ-TCTAAAGGTTGTGGGTCTGC-3’
TGF-β	Forward: 5ʹ-AGGATCAGTAGGTGGAATAG-3’
	Reverse: 5ʹ-TCTAAAGGTTGTGGGTCTGC-3’
β-actin	Forward: 5ʹ-ATGTTGAGACCTTCAACACC-3’
	Reverse: 5ʹ-AGGTAGTCACCTCCCGGCC-3’
miR-30a	Forward: 5ʹ-CCTCCTGCATCCTTTCTTT-3’
	Reverse: 5ʹ-CCTGTCCTTTTTCCTTCC-3’
U6	Forward: 5ʹ-CTCGCTTCGGCAHCACA-3’
	Reverse: 5ʹ-AACGCTTACGAATTTGCGTC-3’

### Western blotting

The total protein of the ovarian cancer cell line was extracted and separated by SDS-PAGE and transferred by a semi-dry method. After blocking with 5% skim milk for 2 h, the membranes were incubated with appropriate primary antibody LC3, Beclin1, TGF-β, Smad4 (dilution, 1:500), and β-actin (dilution, 1:1000) at 4°C overnight. The next day, Alexa Fluor 680 donkey anti-rabbit IgG (H + L) or Fluor 680 donkey anti-mouse IgG (H + L) secondary antibodies (1:5 000) were incubated with membranes at 37°C for 1 h. After washing, the Odyssey^TM^ infrared imaging system was employed to determine the relative expression of target proteins.

### Cell transfection

A2780 and A2780-DDP cells in the logarithmic growth phase were transferred into plates with 96 wells (3 × 10^3^/mL). The miR-30a mimics or mimics NC was transfected into cells using Lipofetamine^TM^ 2000 according to the manufacturer’s instructions. Three different groups were set, including blank control group, negative control group (mimics NC), and miR-30a group (miR-30a mimics).

### CCK8 assay

A2780 and A2780-DDP cells in the logarithmic growth phase were inoculated into 96-well plates (3 × 10^3^ cells/mL) and incubated in medium containing 10% FBS for 24 h, followed by incubation in FBS-free medium containing different concentrations of DDP (0, 1, 5, 10, 15, 20, 50, and 100 μmol/L) for 24 h. Afterward, 10 μL of CCK-8 working solution was added into each well, and the absorbance was measured at 570 nm after incubation for additional 2 h.

The following six groups were set: the untreated cells were used as the blank control group, the cells treated with 10 μmol/L DDP for 48 h were taken as the DDP group, the cells treated with 5 μmol/L autophagy inhibitor 3-MA for 2 h were used as the 3-MA group, the cells treated with 10 μmol/L autophagy activator rapamycin for 2 h were taken as the rapamycin group, the cells treated with 5 μmol/L 3-MA for 2 h and 10 μmol/L of DDP for 48 h were named as the 3-MA+DDP group, and the cells treated with 10 μmol/L rapamycin for 2 h and 10 μmol/L DDP for 48 h were taken as the rapamycin + DDP group. Cell viability (%) was counted based on the following formula: absorbance value of the experimental group/absorbance value of the control group × 100%.

### Flow cytometry

Apoptosis and autophagy levels were assessed by flow cytometry. In brief, A2780 and A2780-DDP cells in the logarithmic growth phase were adjusted to 1 × 10^4^/mL, followed by the preparation of 400 μL of cell suspension using PBS solution. Afterward, 10 μL of horseradish peroxidase-labeled Annexin V (Annexin V-FITC) and iodine propionylation (PI) of 5 μL were added and incubated together for 30 min in dark at room temperature, followed by detection of cell apoptosis rate using flow cytometry.

In terms of autophagy detection, A2780 and A2780-DDP cells were adjusted to 1 × 10^4^/mL, followed by the preparation of 400 μL of cell suspension using PBS solution. After incubation with MDC of 0.05 mmol/L for 45 minutes at a temperature of 37°C and subsequent washing with PBS three times, the autophagy level was assessed by analyzing MDC staining positive cells using flow cytometry. The assay was performed in triplicate. Detection of apoptosis and autophagy was performed following the instruction of the manufacturers.

### Statistical analysis

SPSS 21.0 (IBM, USA) and GraphPad Prism 5.0 (GraphPad Software, USA) statistical software were used for statistical analysis. Data were presented as mean ± standard deviation (SD). To examine normality of the quantitative variables, Shapiro Wilk test was used. Two-sample *t*-test or one-way analysis of variance (ANOVA) was performed to compare the difference between two groups or multiple groups. P < 0.05 was considered statistically significant.

## Results

### MiR-30a was down-regulated, while TGF-β and smad4 were up-regulated in OC after DDP chemotherapy

**Figure 1.**
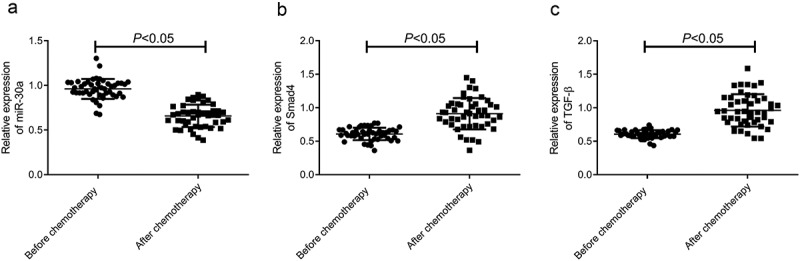
Expression of miR-30a, Smad4, and TGF-β in serum of ovarian cancer patients treated with DDP. A–C, qRT-PCR was used to detect the relative expression level of miR-30a (a) Smad4 (b) and TGF-β (c)

First, we examined the expression of miR-30a, TGF-β, and Smad4 in OC patients before and after DDP chemotherapy using qRT-PCR. As shown in Figure 1a-C, the expression of miR-30a was significantly down-regulated in the serum of DDP chemotherapy patients compared with that before chemotherapy, while the expression of TGF-β and Smad4 was significantly up-regulated (*p* < 0.05).

### Activation of autophagy induced DDP resistance in ovarian cancer cells

**Figure 2.**
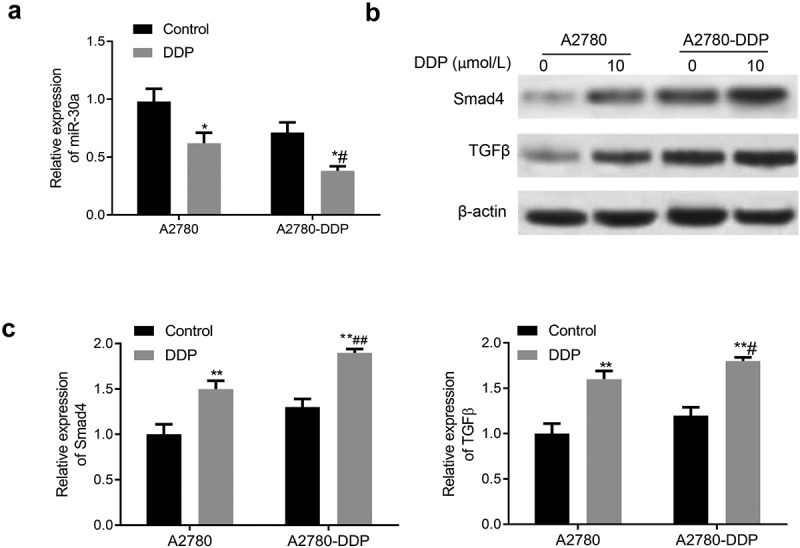
Autophagy promotes DDP resistance in ovarian cancer cells. (a) CCK-8 assay was performed to detect the cell viability of A2780 and A2780-DDP cells. **P < 0.01 *vs*. A2780 group. (b) The protein expression of LC3I/II and Beclin1 were measured by western blotting; (c) CCK-8 assay was used to detect the cell viability; 3-MA, autophagy inhibitor; RAPA, autophagy activator rapamycin; **P < 0.01 vs. Blank control group; #P < 0.05 vs. 3-MA group; &P < 0.05 vs. RAPA group

To determine the relationship between autophagy and DDP-resistance in OC, we first determined the effect of DDP on the viability of OC cells A2780 and A2780-DDP cells. CCK-8 assay revealed that DDP significantly inhibited the growth in A2780 and A2780-DDP cells. A2780-DDP cells were resistant to DDP, whose viability was dramatically higher than the A2780 cells when treated with the same DDP concentration (Figure 2a), indicating the successful construction of DDP resistant cells. From the western blotting, we acknowledged that the LC3 II and Beclin1 expression levels were notably higher in A2780-DDP cells when compared to the A2780 cells regardless of the DDP treatment (Figure 2b). Also, autophagy inhibitor 3-MA promoted DDP-induced apoptosis, while the autophagy activator rapamycin resisted DDP-induced apoptosis (Figure 2c).

### Expression of miR-30a, TGF-β, and smad4 in DDP drug-resistant cells

**Figure 3.**
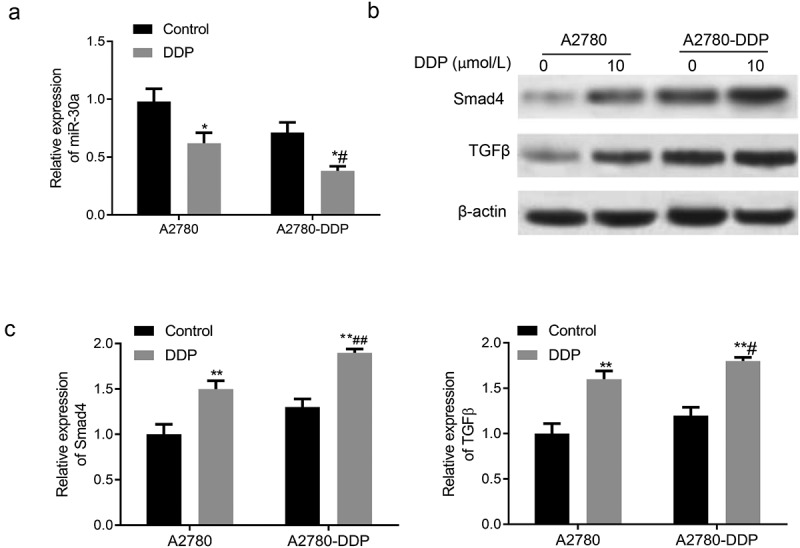
Expression of miR-30a, TGF-β, and Smad4 in DDP drug-resistant cells. (a) The miR-30a expression in A2780-DDP and A2780 cells was detected using qRT-PCR. (b) Western blotting was used to determine the protein expression of Smad4 and TGF-β in A2780 and A2780-DDP cells. (c) qRT-PCR was used to measure the mRNA expression of Smad4 and TGF-β. Compared with the blank control group, *P < 0.05 and **P < 0.01 *vs*. Control group; #P < 0.05 and ##P < 0.01 *vs*. DDP in A2780

Subsequently, we determined the expression of miR-30a, TGF-β, and Smad4 in DDP-resistant OC cells. As shown in Figure 3a, the miR-30a expression in A2780 and A2780-DDP cells was down-regulated after incubation with DDP for 48 h, with more significant down-regulation in the A2780-DDP cells. The treatment of DDP in A2780-DDP and A2780 cells generated significantly enhanced expression of TGF-β and Smad4, with more obvious up-regulation in the A2780-DDP cells (Figure 3b-c).

### MiR-30a inhibits smad4 and TGF-β expression in DDP drug-resistant cells

**Figure 4.**
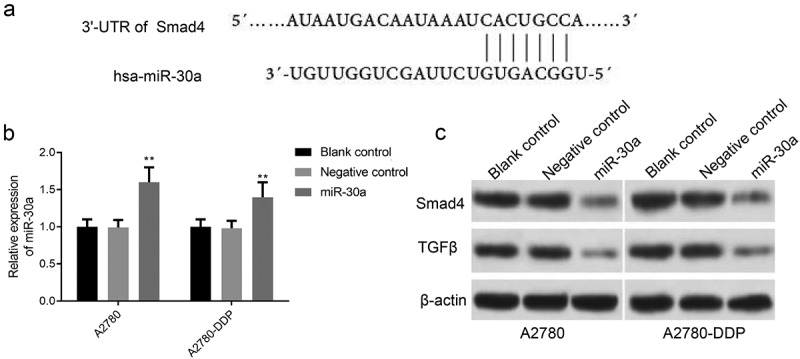
MiR-30a inhibits Smad4 and TGF-β expression in DDP drug-resistant cells. (a) TargetScan analysis indicated that there was a base complement site in miR-30a and Smad4 gene. (b) qRT-PCR detection of miR-30a expression after transfection with miR-30a mimics or mimics NC. **P < 0.01 vs. negative control group. (c) Western blotting was used to determine the protein expression of Smad4 and TGF-β in A2780 and A2780-DDP cells

Using TargetScan software and proteomic analysis, we found that the 3ʹ-UTR of Smad4 gene had a base-complementary site with miR-30a (Figure 4a). As shown in Figure 4b, qRT-PCR results confirmed that miR-30a expression was significantly up-regulated after transfection with miR-30a mimics in A2780 and A2780-DDP cells. Also, the Smad4 and TGF-β expression was down-regulated in miR-30a transfected cells in comparison to the negative control group (Figure 4c).

### MiR-30a suppressed DDP-induced autophagy activation by regulating the TGF-β/smad4 pathway in ovarian cancer cells

**Figure 5.**
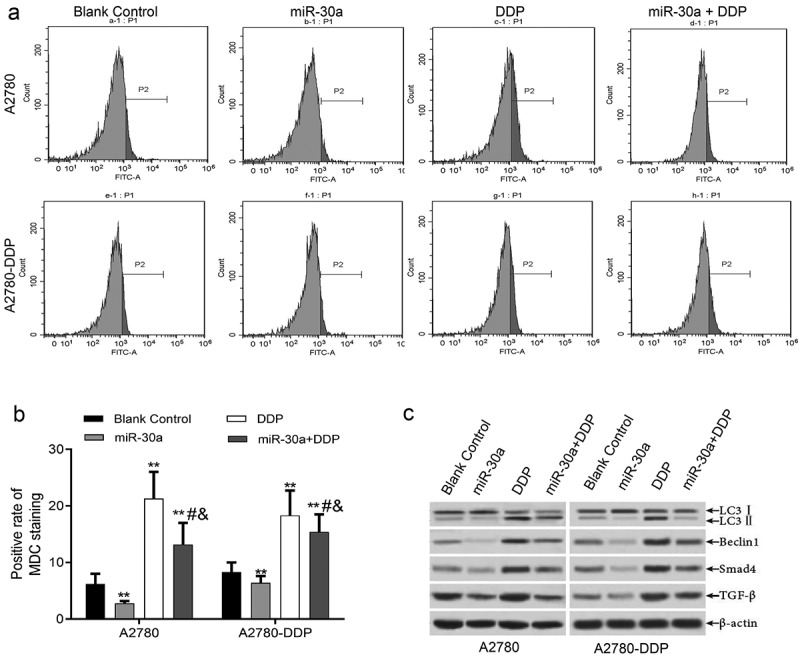
miR-30a suppressed autophagy by regulation of TGF-β/Smad4 signaling pathway. (a-b) MDC staining was detected by flow cytometry, **P < 0.01 vs. Blank control group; #P < 0.05 vs. DDP group; &P < 0.05 vs. miR-30a group. (c) Western blotting detected the expression of autophagy-related proteins (LC3II and Beclin1) and TGF-β/Smad4 pathway proteins

It can be seen from the results that when compared to the blank control group, the MDC positive staining rates in A2780 and A2780-DDP cells of miR-30a transfection group markedly reduced, while that in DDP treatment group apparently increased. In the same cell types, when compared to the DDP group, the MDC positive staining rate of ovarian cancer cells treated with DDP and transfected with miR-30a mimics apparently decreased (Figure 5a-b). Also,western blotting demonstrated that when compared to the blank control group, the LC3II, Beclin1, TGF-β, and Smad4 protein expression in A2780-DDP and A2780 cells in the transfection group of miR-30a was down-regulated, while that of LC3II, Beclin1, TGF-β, and Smad4 protein expression in DDP group was notably up-regulated. In the same cell types, compared with the DDP group, the expression of LC3II, Beclin1, TGF-β, and Smad4 proteins of ovarian cancer cells treated with DDP and transfected with miR-30a mimics was markedly down-regulated (Figure 5c).

### Over-expression of miR-30a suppressed autophagy and promoted DDP-induced apoptosis in ovarian cancer cells

**Figure 6.**
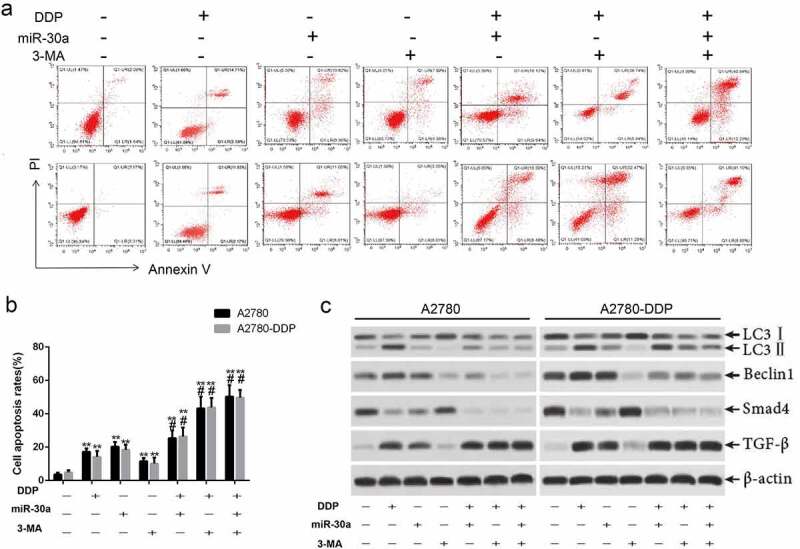
Over-expression of miR-30a suppressed autophagy and promoted DDP-induced apoptosis by inhibition of TGFβ/Smad4 signaling pathway. (a-b) Cells apoptosis was detected by flow cytometry. **P < 0.01 vs. The first group; #P < 0.05 vs. DDP group. (c) Western blotting was used to detect the expression of TGF-β, LC3II, Beclin1, and Smadf4 in A2780 and A2780-DDP cells

We evaluated the effect of the autophagy inhibitors 3-MA and DDP on apoptosis of OC cells in order to study the mechanism of DDP-induced apoptosis of OC cells. The experimental results are presented in Figure 6a-B, suggesting that 3-MA enhances the DDP-induced apoptosis of A2780 and A2780-DDP cells. Western blotting results suggested that 3-MA induced the down-regulation of TGF-β, LC3I/II, Beclin1, and Smad4 in ovarian cancer cells (Figure 6c). Such results revealed that the activation of autophagy might suppress the apoptosis of OC cells induced by DDP, while the miR-30a/TGF-β/Smad4 signaling pathway exerted a regulatory function that was essential during this process.

## Discussion

In the present study, we found that miR-30a levels in peripheral blood were significantly reduced after DDP-based chemotherapy. Further studies have confirmed that miR-30a suppresses the autophagy activation by targeting Smad4 expression. More importantly, autophagy inhibitors promote miR-30a-induced apoptosis and miR-30a enhances chemotherapeutic resistance by inhibiting autophagy in OC cells. A report has confirmed that the TGF-β/Smad4 pathway is essential in the regulation of autophagy [23].

OC is a common malignant tumor in the female genital system with increasing morbidity and mortality. The onset of OC is insidious, and when the patients go to hospital, it has already developed to the advanced stage, with only 5 years for survival even after the surgery. Therefore, chemotherapy is indispensable in treating the OC [24]. Autophagy is important in regulating the cellular life, and its relationship with apoptosis is also a hot topic at present [25,26]. In recent years, it has been reported that autophagy can attenuate the effects of chemotherapy and enhance the chemotherapeutic resistance of tumor cells [27]. Lv et al. [28] has found that the overexpression of CD44v6d gene induces chemotherapeutic resistance in tumor cells by activating autophagy. Another study has reported that autophagy can resist oxaliplatin-induced colorectal cancer stem cell apoptosis and promote cell survival [29]. Autophagy is a potential mechanism to improve chemotherapeutic resistance in tumors and to promote tumor cell survival; therefore, selective inhibition of autophagy is likely to improve chemotherapeutic efficacy. Our present findings have indicated that DDP induces autophagy activation in OC cells, and autophagy protects OC cells against DDP-induced apoptosis.

miRNAs are important in regulating the tumor pathogenesis and progression [30]. Abnormal expression of miRNAs can affect the sensitivity of tumor cells to chemotherapeutic drugs, thereby leading to drug resistance [31]. It has been confirmed that miR-30a is an anti-cancer gene by targeting and inhibiting the expression of different oncogenes, which is closely linked to the drug resistance of tumor cells [32,33]. In this study, we have shown that the miR-30a expression is degenerated in peripheral blood of patients with OC receiving DDP treatment; miR-30a expression is decreased in DDP-resistant OC cells. Also, the exogenous overexpression of miR-30a promotes DDP-induced apoptosis of OC cells.

Among the target genes of miR-30a, Smad4 is essential in regulating the autophagy activation and is a core factor of the TGF-β/Smad pathway [34]. This study has shown that the Smad4 and TGF-β expression increased in peripheral blood of patients with OC after the DDP chemotherapy; miR-30a expression was negatively correlated with the mRNA expression of Smad4, and miR-30a could inhibit the TGF-β and Smad4 expression. Gene software analysis combined with dual luciferase reporter results suggests that miR-30a is able to target and suppress the Smad4 expression in OC cell lines, and the TGF-β/Smad signaling pathway is involved in the regulation of miR-30a-inhibited autophagy and induction of chemotherapeutic resistance against DDP in OC cells to some extent. Exogenous transfection of miR-30a plasmid could decrease chemotherapeutic resistance by inhibiting autophagy in OC cells, thereby increasing the therapeutic efficacy of DDP. Autophagy inhibitor 3-MA enhances the apoptosis levels induced by DDP and exogenously transfects miR-30a.

## Conclusion

Collectively, our present outcomes indicate that miR-30a inhibits autophagy and decreases chemotherapeutic resistance against DDP in OC cells by suppressing the TGF-β/Smad4 pathway’s activation, thereby improving the efficacy of DDP clinical treatment of OC.

## Highlights

(1) MiR-30a was down-regulated in DDP-resistant ovarian cancer cells.

(2) Activation of autophagy contributed to DDP-resistance cells.

(3) Overexpression of miR-30a decreased the expression of Smad4 and TGF-β.

(4) MiR-30a-overexpressing inhibited DDP-induce autophagy and promoted DDP-resistant cells apoptosis.
